# 

**Published:** 1891-09

**Authors:** 


### Bristol Medico-Chirurgical Journal.

SEPTEMBER, 1891.

### CONTENTS.

ORIGINAL PAPERS: *Page*

Chloroform *versus* Ether. By W. H. HARSANT, F.R.C.S. Eng.... ... 145

Cases of Septic Thrombosis of the Lateral Sinus. By CHARLES F. PICKERING, F.R.C.S. Eng. ... ... ... ... ... ... ... ... ... ... 155

Case of Tumour of the Pons. By P. WATSON WILLIAMS, M.B. Lond. 163

Buxton: Its Baths and Climate. By ALFRED J. H. CRESPI, M.R.C.S. Eng., M.R.C.P.I. ... ... ... ... ... ... ... ... ... ... ... ... ... 167

PROGRESS OF THE MEDICAL SCIENCES ... ... ... ... ... ... ... ... 184

REVIEWS OF BOOKS ... ... ... ... ... ... ... ... ... ... ... ... ... ... 204

NOTES ON PREPARATIONS FOR THE SICK ... ... ... ... ... ... ... ... 213

THE LIBRARY OF THE BRISTOL MEDICO-CHIRURGICAL SOCIETY ... ... ... 215

SCRAPS ... ... ... ... ... ... ... ... ... ... ... ... ... ... ... ... ... 238

---

*Cases for binding, price 7d. each, may be had of the Publisher,*

J. W. ARROWSMITH, *Quay Street, Bristol.*

### The Library of the

### Bristol Medico-Chirurgical Society.

*The following is a List of Donations received up to August 31st, 1891.*

| | £ | s. | d. | | | |
|---|---|---|---|---|---|---|
| W. R. Ackland ... ... ... | 20 | 0 | 0 | | | |
| J. W. Arrowsmith (1) ... ... | | | | ... | 1 | volume. |
| Barclay J. Baron, M.B. (2) ... | | | | ... | 2 | volumes. |
| John Beddoe, M.D. (3) ... | | | | ... | 61 | ,, |
| A. E. Blacker (4) ... ... | | | | ... | 5 | ,, |
| British Medical Association (5) | | | | ... | 706 | ,, |
| John Broom, M.D. (6) ... | | | | ... | 8 | ,, |
| J. Michell Clarke, M.B. (7) ... | | | | ... | 17 | ,, |
| G. G. Corbould (8) ... ... | 5 | 0 | 0 | and | 33 | ,, |
| W. Howard Cory (9) ... ... | | | | ... | 3 | ,, |
| F. R. Cross (10) ... ... | 10 | 0 | 0 | and | 26 | ,, |
| D. S. Davies, M.B. (11) ... | | | | ... | 2 | ,, |
| Nelson C. Dobson ... ... | 5 | 0 | 0 | | | |
| T. S. Ellis (12)... ... ... | | | | ... | 1 | volume. |
| John Ewens (13)... ... ... | | | | ... | 4 | volumes. |
| E. Long Fox, M.D. (14) ... | 20 | 0 | 0 | and | 23 | ,, |
| W. J. Fyffe, M.D. (15) ... | 5 | 0 | 0 | and | 6 | ,, |
| L. M. Griffiths (16) ... ... | 5 | 5 | 0 | and | 54 | ,, |
| A. J. Harrison, M.B. ... ... | 5 | 0 | 0 | | | |
| F. P. Lansdown (17) ... ... | | | | ... | 59 | ,, |
| Henry Marshall, M.D. ... ... | 2 | 2 | 0 | | | |
| C. A. Morton ... ... ... | 0 | 10 | 0 | | | |
| New York Academy of Medicine (18) ... ... ... ... | | | | ... | 6 | ,, |
| George Parker, M.D. ... ... | 2 | 2 | 0 | | | |
| Mrs. J. D. F. Parsons (19) ... | | | | ... | 27 | ,, |

| | £ | s. | d. | | |
|---|---|---|---|---|---|
| W. J. Penny (20) ... ... | 1 | 1 | 0 | and | 3 volumes. |
| Young J. Pentland (21)... ... | | | | ... | 1 volume. |
| C. F. Pickering (22) ... ... | | | | ... | 1 ,, |
| Augustin Prichard ... ... | 5 | 0 | 0 | | |
| Arthur B. Prowse, M.D. (23) | | | | ... | 47 volumes. |
| J. Morgan Richards (24) ... | | | | ... | 1 volume. |
| R. B. Ruddock (25) ... ... | | | | ... | 194 volumes. |
| C. King Rudge (26) ... ... | | | | ... | 1 volume. |
| E. J. Sheppard ... ... ... | 1 | 1 | 0 | | |
| J. Greig Smith (27) ... ... | | | | ... | 62 volumes. |
| R. Shingleton Smith, M.D. (28) | 5 | 5 | 0 | and | 24 ,, |
| Surgeon-General, U.S. Army (29) | | | | ... | 6 ,, |
| J. G. Swayne, M.D. (30) ... | | | | ... | 107 ,, |
| Vandenhoeck & Ruprecht (31) | | | | ... | 3 ,, |

Unbound periodicals have been received from Dr. Beddoe, The British Medical Association, Dr. Broom, Mr. Corbould, Mr. Dobson, Mr. Griffiths, Mr. Lansdown, and Mr. Greig Smith.

Framed pictures have been presented by Mrs. Frederick Brittan, Mr. John Dacre, Dr. J. G. Davey, Dr. Long Fox, Dr. Fyffe, Mr. Griffiths, Mr. Augustin Prichard, Dr. Barrett Roué, and Mr. S. H. Swayne.

---

### LIST OF BOOKS.

The figures in brackets refer to the figures after the names of the donors, and show by whom the volumes were presented. The books to which no such figures are attached have either been bought from the Library Fund or received through the *Journal.*

The list of books makes no pretension of bibliographical fulness. It is merely a record of short titles supplied as a matter of convenience.

---

**Abbott, J.** ... *Hand Book of Idiotcy* ... ... ... ... ... ... ... (5) 1856

**Abercrombie, J.** *On Diseases of the Stomach* ... ... (5) 3rd Ed. 1837

**Abernethy, J....** *The Origin and Treatment of Local Diseases, and on Aneurisms.* (25) 1809. (5) 1815.
(19) 5th Ed. 1820. (3) 6th Ed. 1822. (30) 7th Ed. 1824

,, *On Injuries of the Head—*
(25) 1810. (5) 1815. (19, 30) 3rd Ed. 1821

**Abernethy, J....** *On Diseases resembling Syphilis.* (25) 1810. (19) 3rd Ed. 1814. (5) 1815. (30) 4th Ed. 1822
,, *On Tumours and on Lumbar Abscesses.* (25) 1811. (5) 1815. (19) 2nd Ed. 1816. (30) 3rd Ed. 1822
,, *Anatomical Lectures* ... ... ... ... ... ... ... (25) 1814
,, *Lectures on Surgery, &c.* ... ... ... (25) 2nd Ed. 1831
**Acland, H. W.** *The Harveian Oration,* 1865 ... ... ... ... (5) 1865
**Acton, W.** ... *The Reproductive Organs* ... ... ... (25) 3rd Ed. 1862
**Adams, J.** ... *On the Prostate Gland* ... ... ... ... ... ... ... (25) 1851
**Addison, T.** ... *Works* (Ed. by Dr. Wilks and Dr. Daldy) (5) 1868
**Agnew, D. H....** *Surgery.* 3 vols. ... ... ... ... ... ... (10) 1878-83
**Aldridge, J.** ... *On the Urine* ... ... ... ... ... ... ... ... ... (25) 1846
**Allan, F. J.** ... *Aids to Sanitary Science*... ... ... ... ... ... ... ... 1890
**Allen, H.** ... *A New Method of Recording the Motions of the Soft Palate* ... ... ... ... ... ... ... ... ... (10) 1884
**Amesbury, J....** *Observations on Fractures* ... ... ... ... ... ... ... 1828
,, *Fractures.* 2 vols. ... ... ... ... ... ... ... (30) 1831
* **Andral, G.** ... *Clinique médicale.* (3) Tomes I.—IV. 4th Ed. 1839-40; (3) Tome V. 2nd Ed. 1833
,, *Précis d'Anatomie pathologique.* 3 vols. ... ... (30) 1829
*Aneurism, Observations on.* (Tr. and Ed. by J. E. Erichsen) ... (5) 1844
**Aretæus** (The Cappadocian). *Extant Works*—Greek and English. (Ed. and Tr. by F. Adams) ... ... ... ... ... (5) 1856
* **Arnott, N.** ... *Elements of Physics* ... ... (25) Vol. I. 5th Ed. 1833
**Ashhurst, J.**[Ed.] *The International Encyclopædia of Surgery.* 6 vols. (10) 1882-86
**Ashton, T. J.** ... *Prolapsus, Fistula in Ano and Hæmorrhoids* ... (25) 1862
**Ashwell, S.** ... *Diseases of Women* ... ... ... ... ... ... ... (30) 1844

**Baas, J. H.** ... *History of Medicine* ... ... ... ... ... ... ... ... 1889
**Baillie, M.** ... *Morbid Anatomy.* (Ed. by J. Wardrop.) (25) 6th Ed. 1830
**Bainbrigge, —.** *Droitwich Salt Baths* ... ... ... ... ... ... ... (25) 1873
**Ball, J. B.** ... *Diseases of the Nose* ... ... ... ... ... ... ... (2) 1890
,, *Intubation of the Larynx* ... ... ... ... ... ... ... 1891
**Ballantyne, J. W.** *Introduction to the Diseases of Infancy*... .. ... ... 1891
**Ballard, E.** ... *Pain after Food* ... ... ... ... ... ... ... ... (25) 1854
**Barbour, D. B. Hart and A. H. F.** *Manual of Gynecology.* (5) 3rd Ed. 1886
**Barry, F. W....** *Report of an Epidemic of Small Pox at Sheffield,* 1887–88 (16) 1889
**Barthez et F. Rilliet, A. C. E.** *Maladies des Enfants.* 3 vols. (30) 1843
**Bartholinus, T.** *Anatome* ... ... ... ... ... ... ... ... ... 5th Ed. 1686
**Basham, W. R.** *Dropsy connected with Disease of the Kidneys.* (25) 1858
**Bateman, T.** ... *On Cutaneous Diseases* ... ... ... ... (19) 6th Ed. 1824
**Beale, L....** ... *The Examination of Urine* ... ... ... ... ... (25) 1856
**Beasley, H.** ... *Book of Prescriptions* ... ... ... ...(23) 2nd Ed. 1859

**Beck, T. R.** ... *Medical Jurisprudence.* (5) 2nd Ed. 1825.
(30) 6th Ed. (conjointly with J. B. Beck) 1838
**Beck, T. R. Beck and J. B.** *Medical Jurisprudence.* (30) 6th Ed. 1838
**Beddoe, J.** ... *The Races of Britain* ... ... ... ... ... ... (1) 1885
**Bell, B.** ... ... *A System of Surgery.* 6 vols.
(5) Vol. I. 1783; (5) Vol. II. 1784;
(5) Vol. III. 1785, (5) 3rd Ed. 1788;
(5) Vol. IV. 1786, (5) 2nd Ed. 1787;
(5) Vol. V. 3rd Ed. 1789; (5) Vol. VI. 3rd Ed. 1789
**Bell, C.** ... ... *The Nervous System of the Human Body* ... ... (5) 1830
**Bell, J.** ... ... *Operations of Surgery* ... ... ... ... (5) 3rd Ed. 1874
,, *Notes on Surgery for Nurses* ... ... ... 3rd Ed. 1891
* **Bell, J. and C.** *Anatomy and Physiology of the Human Body.*
(5) Vols. II., III. 5th Ed. 1823
**Bellinus, L.** ... *Opuscula & Exercitationes.* Two vols. in one. (3) 1714–26
**Bennet, J. H.** ... *Inflammation of the Cervix Uteri* ... ... ... (25) 1845
**Bennett, Sir R.** *The Diseases of the Bible* ... ... ... ... ... (16) 1887
**Bennett, W. H.** *Varicocele* ... ... ... ... ... ... ... ... ... ... 1891
**Bernutz and E. Goupil, G.** *Diseases of Women.* (Tr. by A. Meadows)
2 vols. ... ... ... ... ... ... ... ... ... (5) 1866–67
**Berry, G. A.** ... *Diseases of the Eye* ... ... ... ... ... ... ... (16) 1889
,, ... *Ophthalmoscopic Diagnosis* ... ... ... ... ... ... 1891
**Bigg, R. H.** ... *Spinal Curvature* ... ... ... ... ... (23, 25, 30) 1882
,, *Artificial Limbs* ... ... ... ... ... (3, 23, 25) 1885
**Billing, A.** ... *Principles of Medicine* ... ... ... ... (25) 3rd Ed. 1838
**Billings, J. S.** ... *National Medical Dictionary.* 2 vols. ... ... ... 1890
**Billroth, T** ... *Surgery.* 2 vols. ... ... ... ... ... ... (5, 28) 1877–78
**Bird and C. Brooke, G.** *Natural Philosophy* ... ... ... (5) 5th Ed. 1860
**Black, D. C.** ... *The Germ-Theory of Disease*... ... ... ... ... [1891]
**Boerhaave, H.** *Treatise on the Powers of Medicines* ... ... ... (8) 1740
**Bouchut, E.** ... *Diseases of Children.* (Tr. by P. H. Bird) ... (30) 1855
**Bouillaud, J.** ... *Clinique médicale.* 2 vols. ... ... ... ... ... (3) 1837
**Bourneville and Bricon, Drs.** *Hypodermic Medication.* 2nd Ed.
(Tr. by A. S. Currie) ... ... ... ... ... ... (16) 1887
**Bowles, R. L.** ... *Stertor and Apoplexy* ... ... ... ... ... ... ... 1891
**Bowman, J. E.** *Medical Chemistry* ... ... ... ... ... (23) 3rd Ed. 1855
**Boyer, Baron**... *Traité des Maladies chirurgicales.* 12 vols. ... 1822–28
**"B. P. C."** ... *Unofficial Formulary, with Addendum* ... ... ... ... 1889
**Brachet, L.** ... *Aix-les-Bains* ... ... ... ... ... ... ... ... (5) 1884
**Bramwell, B.** ... *Studies in Clinical Medicine* ... ... ... ... Vol. I. 1890
,, *Atlas of Clinical Medicine* ... ... Vol. I. Part 1 1891
**Braun, J.** ... *Curative Effects of Baths and Waters* ... ... ... (5) 1875
**Bricon, Drs. Bourneville and** *Hypodermic Medication.* 2nd Ed.
(Tr. by A. S. Currie) ... ... ... ... ... ... 1887

**Bright, R.** ... *Clinical Memoirs on Abdominal Tumours.* (Ed. by G. H. Barlow) ... ... ... ... ... ... ... ... (5) 1860

**Briot, P. F.** ... *Histoire de l'Etat et des Progrès de la Chirurgie militaire en France* ... ... ... ... ... ... ... (5) 1817

**Bristowe, J. S.** *Theory and Practice of Medicine* ... (5) 5th Ed. 1884

**Brodie, B. C.** ... *On Diseases of the Joints* ... ... ... ... ... ... (5) 1818

,, *On the Urinary Organs.* (5) 3rd Ed. 1842. (3) 4th Ed. 1849

**Brooke, G. Bird and C.** *Natural Philosophy* ... ... ... (5) 5th Ed. 1860

**Brown, I. B.** ... *On Diseases of Women* ... ... ... ... ... ... ... (5) 1854

**Browne, L.** ... *Koch's Remedy in Throat Consumption*.. ... ... ... 1891

**Brown-Séquard, E.** *Researches on Epilepsy* ... ... ... ... ... ... (23) 1857

**Bruce, J. M.** ... *Materia Medica and Therapeutics.* (16) 3rd Ed. 1886 1891

**Brunton, T. L.** *Pharmacology, Therapeutics and Materia Medica.* 3rd Ed. 1887

**Bryant, T.** ... *Harveian Lectures* ... ... ... ... ... ... ... ... (23) 1885

**Buck, A. H.** [Ed.] *Reference Handbook of the Medical Sciences.* 8 vols. 1888–90

**Bull, T.** ... ... *Hints to Mothers* ... ... ... ... ... ... (25) 2nd Ed. 1839

**Burnet, R. W.** *Foods and Dietaries* .. ... ... ... .. ... ... 1890

**Burns, J.** ... *Principles of Midwifery.* (19) 6th Ed. 1824. (25) 9th Ed. 1837

*Bushe on Diseases of the Rectum, Plates to* ... ... .. ... ... ... (9) 1837

**Butlin, H. T.**... *Diseases of the Tongue* ... ... ... ... ... ... ... (5) 1885

**Caillard, E. M.** *Invisible Powers of Nature* ... ... ... ... ... ... (5) 1888

**Caird and C. W. Cathcart, F. M.** *Students' Atlas of Bones and Ligaments* ... ... ... ... ... ... ... .. ... ... 1885

**Cameron, Sir C. A.** *History of the Royal College of Surgeons in Ireland* (5) 1886

**Campbell, H.** ... *Differences in the Nervous Organisation of Man and Woman* ... ... ... ... ... ... ... ... ... ... 1891

**Carlsen, J.** ... *Statistiske Undersogelser angaaende Aandssvage i Danmark.* 1888–1889. (With Abstract in English) 1891

**Carter, A. H.** ... *Elements of Medicine.* (16) 5th Ed. 1888. 6th Ed. 1891

**Carter, R. B.** ... *Hysteria* ... ... ... ... ... .. ... ... (25, 30) 1853

**Casper, J. L.** ... *Forensic Medicine.* (Tr. by G. W. Balfour.) (5) Vols. I.—III. 1861–64

*Catalogue of Library of Royal Medical and Chirurgical Society.* (5) *Index*, 1860: 3 vols. (5) 1879

*Catalogue (Index) of Library of Surgeon-General's Office, U.S. Army.* Vols. I.—V. 1880–84. (29) Vols. VI.—XI. 1885–90

*Catalogue of Obstetric Instruments, &c.* ... ... ... ... ... ... ... (8) 1867

**Cathcart and F. M. Caird, C. W.** *Students' Atlas of Bones and Ligaments* ... ... ... ... ... ... ... ... ... ... 1885

**Chambers, T. K.** *Digestion and its Derangements* ... ... ... ... (25) 1856

,, *The Renewal of Life* .. .. ... ... (25) 2nd Ed. 1863

**Champneys, F. H.** *Painful Menstruation* ... ... ... ... ... ... ... ... 1891

* **Charcot, J. M.**... *Œuvres complètes* ... ... ... ... ... ... (5) Tome V. 1888

**Chaumont, F. S. B. F. de** *Lectures on State Medicine* ... ... ... ... (15) 1875

**Chelius, J. M.**... *System of Surgery.* (Tr. by J. F. South.) 2 vols. (23) 1847
**Cheyne, W. W.** *Suppuration and Septic Diseases* .. ... ... ... ... ... 1889
*Child's First Hour, A* ... ... ... ... ... ... ... .. ... ... ... (25) 1851
**Chisholm, J. E. Pollock and J.** *Medical Handbook of Life*
,, *Assurance* ... . ... ... ... ... ... ... 3rd Ed. 1889
**Churchill, F.** ... . *Diseases of Children* ... ... ... ... ... ... ... (25) 1850
,, *Diseases of Women*... ... ... ... ... ... 4th Ed. (30) 1857
**Clarke and C. M. Tidy, P.** *Medical Laws for Medical Men* ... ... ... 1890
* *Clinical Lectures, German.* Second Series ... ... ... ... ... ... (5) 1877
**Cloquet, H.** ... *Anatomy.* (Tr. by R. Knox) . . ... ... ... ... (5) 1828
**Cloquet, J.** ... *On Hernia.* (Tr. by A. M. McWhinnie)... ... (25) 1835
**Coates, W. M.**... *On Chloroform* ... ... ... ... ... ... ... ... (25) 1858
**Coats, J.** ... ... *Manual of Pathology* ... ... ... ... ... ... 2nd Ed. 1889
**Colles, A.** ... *Works.* (Ed. by R. McDonnell) ... ... ... ... (5) 1881
**Combe, A.** ... *The Management of Infancy* ... ... ... ... (5) 9th Ed. 1860
**Conolly, J.** ... *On the Indications of Insanity* ... ... ... ... ... (19) 1830
,, *The Construction and Government of Lunatic Asylums.* (19) 1847
**Cooke, T.**... ... *Tablets of Anatomy* ... ... ... ... ... (10) 4th Ed. 1885
**Cooper, Sir A.**... *Dislocations and Fractures*— (22) 4th Ed. 1824. (5) 7th Ed. 1831
,, *Lectures on Surgery, &c.* (25) 3rd Ed. 1832. (5) 6th Ed. 1839
**Cooper, S.** ... *Dictionary of Practical Surgery* ... (5, 19) 4th Ed. 1822
,, *First Lines of Surgery*— (5, 25, 30) 6th Ed. 1836. (5) 7th Ed. 1840
**Coote, H.** ... *On Syphilis* ... ... ... ... ... ... ... ... ... (25) 1857
,, *On Joint-Diseases* ... ... ... ... ... ... ... ... ... (3) 1867
**Copeman, E.** ... *Records of Obstetric Practice* ... ... ... ... ... ... (30) 1856
**Copland, J.** ... *Medical Dictionary.* 4 vols. ... ... (13, 23) 1844–58
**Coppock, J. Craven and T.** *The Law Relating to Medical Practitioners* 1890
**Cormack, C. E.** *The Mineral Waters of Vichy* ... ... ... ... (5) 1887
**Cotton, R. P.** ... *Phthisis and the Stethoscope* ... ... ... ... ... ... (25) 1851
**Coupland, S.** ... *Notes on the Examination of the Sputum, Vomit, Fæces, and Urine* ... ... ... ... ... ... ... 1891
**Cox, F. A.** ... *The Care of the Skin* ... ... ... ... ... ... ... (16) 1890
**Craven and T. Coppock, J.** *The Law Relating to Medical Practitioners.* 1890
**Critchett, A.** ... *Eclecticism in Operations for Cataract* ... ... ... (23) 1883
**Crocker, H. R.**... *Diseases of the Skin* ... ... ... ... ... ... ... (16) 1888
**Crookshank, E. M.** *Manual of Bacteriology* ... ... ... (27) 3rd Ed. 1890
**Cullen, W.** ... *Materia Medica.* 2 vols. ... ... ... ... ... ... (30) 1789
,, *Practice of Physic.* (Ed. by J. C. Gregory.) 2 vols. (19) 1829
**Cullimore, D. H.** *Consumption as a Contagious Disease* ... ... (15) [1880]
**Cummin, W.** ... *The Proofs of Infanticide* ... ... ... ... ... ... (25) 1836
**Curgenven, J. B.** *Eucalyptus Globulus in Scarlet Fever* ... ... ... ... 1891
**Curling, T. B.**... *Diseases of the Testis.* (5) 1843. (8) 2nd Ed. 1856

**Czermak, J. N.** *Practical Uses of the Laryngoscope.* (Translated by G. D. Gibb) ... ... .. ... ... ... (3, 5) 1861

**Daventer, H. à** *The Art of Midwifery Improv'd* ... (5) 3rd Ed. 1728
**Davis, D. D.** ... *Obstetric Medicine.* 2 vols. ... ... ... ... ... (5) 1836
**Davis, J. H.** ... *On Difficult Parturition* ... ... ... ... ... ... (5) 1858
**Dawson, R.** ... *Spermatorrhœa.* (3) 5th Ed. 1851. (30) 6th Ed. 1852
*Denmark: its Medical Organization, Hygiene and Demography* ... ... 1891
**D'Estrées, D.** ... *Medical Guide to Contrexéville* ... .. ... ... (5) 1883
**Dewees, W. P.** *Midwifery* ... ... ... ... .. ... ... .. (19) 1825
*Dictionary, English and Italian* (J. E. Wessely) ... ... ... ... [N.D.]
*Dictionary, English and Spanish* (J. E. Wessely and A. Gironés.) 5th Ed. 1888
*Dictionary, French:* Abridged (L. Contanseau) ... ... ... ... [N.D.]
*Dictionary, German-English and English-German* (F. W. Longman.) 6th Ed. 1890
*Dictionnaire de Médecine.* 21 vols. ... ... ... ... ... ... ... ... 1821-28
**Diday, P.** ... *Infantile Syphilis.* (Tr. by G. Whitley) ... ... (16) 1859
*Diphtheria, Memoirs on.* (Tr. by R. H. Semple) ... ... ... ... (5) 1859
**Dobell, H.** ... *Loss of Weight, Blood-spitting, and Lung Disease.* (5) 1878
**Donders, F. C.** ... *Accommodation and Refraction of the Eye.* (Tr. by W. D. Moore) ... ... ... .. ... ... ... (5) 1864
**Doran, A. H. G.** *Tumours of the Ovary, &c.* ... ... ... ... ... (5) 1884
**Downing, C. T.** *Neuralgia* ... ... ... ... ... ... ... ... (30) 1851
**Dowse, T. S.** ... *Massage and Electricity* ... ... ... ... ... (16) [N.D.]
**Druitt, R.** ... *The Surgeon's Vade-Mecum* ... ... (5) 11th Ed. 1878
**Dukes, C.** ... *The Preservation of Health* ... ... ... ... (5) [N.D.]
**Duncan, J. M.** ... *Researches in Obstetrics* ... ... ... ... ... ... ... 1868
,, *On Perimetritis and Parametritis* ... ... ... ... ... ... 1869
,, *Mortality of Childbed and Maternity Hospitals* ... ... 1870
,, *Fecundity, Fertility, and Sterility* ... ... ... 2nd Ed. 1871
,, *Mechanism of Natural and Morbid Parturition* ... (5) 1875
,, *Clinical Lectures on the Diseases of Women* (5) 3rd Ed. 1886
**Dupuytren, Baron** *Diseases and Injuries of Bones.* (Tr. and Ed. by F. le Gros Clark) ... ... ... ... ... ... (5) 1847
,, *Lesions of Vascular System.* (Tr. and Ed. by F. le Gros Clark) ... ... ... ... ... ... (5) 1854
**Dusch, T. v.** ... *Thrombosis of the Cerebral Sinuses.* (Tr. by G. Whitley) ... ... ... ... ... ... ... ... (3, 5) 1861

*Edinburgh Practice.* (5) Vols. I., II. Medicine; (5) Vols. III., IV. Surgery; (5) Vol. V. Midwifery 1803
**Edinger, L.** ... *Structure of the Central Nervous System.* 2nd Ed. (Tr. by W. H. Vittum) ... ... ... ... ... ... 1890
**Ellis, G. V.** ... *Dissection of the Human Body.* 2 vols. Plates and Letterpress ... ... ... ... ... ... ... ... (7) 1867

**Ellis, T. S.** ... *The Human Foot* ... ... ... ... ... ... ... ... (12) 1889
**Emmet, T. A.**... *The Principles and Practice of Gynæcology.* (30) 2nd Ed. 1880
**Erichsen, J. E.** *Science and Art of Surgery.* 2 vols. (5) 7th Ed. 1877
**Esmarch, F.** ... *The Uses of Cold in Surgical Practice.* (Tr. by E. Montgomery) ... ... ... ... ... ... (3, 5) 1861
**Evanson and H. Maunsell, R. T.** *On Diseases of Children.* (5) 1836; (5) 3rd Ed. 1840
*Extra Physics*... ... ... ... ... ... ... ... ... ... ... ... ... ... (5) 1878
**Eyre, Sir J.** ... *On some Exhausting Diseases* ... (25) 2nd Ed. 1851

**Fabricius, G.** (Hildanus) *Opera Observationum et Curationum Medico-Chirurgicorum* ... ... ... ... ... ... (8) 1646

**Faithhorn, J.** .. *On Liver Complaints* ... ... ... ... (3) 5th Ed. 1823

**Falconer, R. W.** *The Baths of Bath* ... ... ... ... (25) 3rd Ed. 1860

**Felkin, R. W.**... *Hypnotism* ... ... ... ... ... ... ... ... 1890

**Fenwick, E. H.** *Electric Illumination of the Bladder and Urethra.* (5) 2nd Ed. 1889

**Fenwick, S.** ... *Medical Diagnosis* ... ... ... ... ... ... (5) 4th Ed. 1876

**Ferguson, F. W. N. Haultain and J. H.** *Handbook of Obstetric Nursing* ... ... ... ... ... ... ... ... ... (16) 1889

**Fergusson, W.** *Practical Surgery* ... ... ... ... (23) 2nd Ed. 1846

**Ferrier, D.** ... *Cerebral Localisation* ... ... ... ... ... ... ... ... 1890

**Feuchtersleben, Baron** *Medical Psychology.* (Tr. by H. E. Lloyd and Ed. by B. G. Babington) ... ... (5) 1847

**Finlayson, J.** ... *The Life and Works of Maister Peter Lowe* ... (5) 1889

**Forbes, J.** ... *A Manual of Medical Bibliography* ... ... ... (25) 1835

**Forsyth, J. S.**... *Medical Dieteticon* ... ... ... ... ... ... ... (3) 1824

**Foster, B.** ... *Clinical Medicine* ... ... ... ... ... ... ... (15) 1874

* **Foster, M.** ... *Text-Book of Physiology.* (5) Parts I., II. 5th Ed. 1888-89

**Fothergill, J. M.** *The Town Dweller* ... ... ... ... ... ... ... (16) 1889

**Fowler, J. K.** [Ed.] *Dictionary of Practical Medicine* ... ... ... ... 1890

**Fox, F.** ... ... *Strathpeffer Spa* ... ... ... ... ... ... ... ... (5) 1889

**Fox, T.** ... ... *Skin Diseases* ... ... ... ... ... ... ... ... (25) 1864

**Fox, W.** ... ... *Diagnosis and Treatment of Dyspepsia*... ... ... (3) 1867

**Frerichs, F. T.** *Clinical Treatise on Diseases of the Liver.* (Tr. by C. Murchison.) 2 vols. ... ... ... (5) 1860-61

**Fuller, A.** ... *South Africa as a Health Resort* ... ... ... ... (5) 1886

* **Fyfe, A.** ... ... *A Compendium of Anatomy.* (5) Vols. II., III. 5th Ed. 1812

**Gant, F. J.** .. *The Irritable Bladder* ... ... ... ... (25) 2nd Ed. 1867

**Garrett, J. H.**... *Action of Water on Lead* ... ... ... ... ... ... 1891

**Garrod, A. B.**... *Materia Medica and Therapeutics* ... ...(25) 3rd Ed. 1868

**Gerrard, A. W.** *New Official Remedies B.P.* 1890 ... ... ... ... 1891

**Gibert, C. M.** ... *Diseases of the Skin.* 2nd Ed. (Tr. by E. Sheppard.) (30) 1845

**Glasgow, Medico-Chirurgical Society of.** *Discussion on Anæsthetics* ... 1891
**Gooch, R.** ... *On Diseases of Women and Children.* (Prefatory Essay by R. Ferguson.) ... ... ... ... ... ... (5) 1859
* **Good, J. M.** ... *Study of Medicine.* (By S. Cooper.) (19) Vols. I.—IV. 3rd Ed. 1829
**Gottstein, J.** ... *Diseases of the Larynx.* (Tr. and added to by P. M'Bride) ... ... ... ... ... ... ... [N.D.]
**Gould, G. M.** ... *Medical Dictionary* ... ... .. ... ... ... ... 1890
**Goupil, G. Bernutz and E.** *Diseases of Women.* (Tr. by A. Meadows.) 2 vols. ... ... ... ... ... (5) 1866-67
**Gowers, W. R.** *Medical Ophthalmoscopy*... .. ... ... ... 3rd Ed. 1890
**Graefe, A. v.** ... *On Iridectomy.* (Tr. by T. Windsor) ... ... (5) 1859
**Gray, H.** ... ... *Anatomy* ... ... ... ... ... ... ... ... ... ... (5) 1858
**Green, J.** ... ... *Diseases of the Skin* ... ... .. ..(25) 2nd Ed. 1837
**Green, T. H.** ... *Pathology and Morbid Anatomy* ... ... (5) 2nd Ed. 1873
* **Gregory, G.** ... *Practice of Physic* ... ... ... ... ... (5) Vol. II. 1823
**Gregory, J.** ... *Conspectus Medicinæ Theoreticæ.* (19) Editio Nona 1832
**Griesinger, W.** *On Mental Diseases.* (Tr. by C. L. Robertson and J. Rutherford) ... ... ... ... ... ... ... (5) 1867
**Griffiths, W. H.** *Materia Medica and Pharmacy* ... ... ... ... (5) 1879
**Groenevelt, J.**... *Dissertatio Lithologica* ... ... ... ... ... 2nd Ed. 1687
**Grove, Sir W. R.** *The Correlation of Physical Forces* ... (8) 6th Ed. 1874
**Guthrie, G. J.**... *Wounds and Injuries of the Chest* ... ... ... (30) 1848
**Guttmann, P.**... *Handbook of Physical Diagnosis.* (Tr. by A. Napier.) (5) 1879
**Guy, W. A.** ... *Public Health* ... ... ... ... ... ... ... ... (10) 1870
,, *The Harveian Oration,* 1875 ... ... ... ... ... (3) 1875

**Habershon, S. O.** *The Effects of Mercury* ... ... ... ... ... ... (25) 1860
,, *The Harveian Oration.* June 27, 1883 ... ... (3) [N.D.]
**Hall, M.** ... ... *The Principles of Diagnosis* ... ... ... (25) 2nd Ed. 1834
* **Hallerus, A.** ... *Disputationes ad Morborum Historiam et Curationem facientes.* ... ... ... ... (8) Vols. I.–VI. 1757–58
,, *Disputationes Chirurgicæ Selectæ.* 5 vols. ... ... 1755–56
**Hamilton, F. H.** *A Treatise on Fractures and Dislocations.* (5) 5th Ed. 1875
**Hamilton, J.** ... *On Purgative Medicines* ... ... ... (25) 2nd Ed. 1806
**Hare, H. A.** ... *Fever* ... ... ... ... ... ... ... ... ... ... 1891
**Harley, G.** ... *Diabetes* ... ... ... ... ... ... ... ... ... (25) 1866
**Harris and D. Power, V. D.** *Physiological Laboratory.* (28) 4th Ed. 1888
**Harrison, J.** ... *Venereal Diseases* ... ... ... ... ... ... ... (25) 1860
**Harrison, R.** ... *Anatomy of the Arteries.* 2 vols. (19) 3rd Ed. 1833
**Hart, D. B.** ... *Female Pelvic Anatomy* ... ... ... ... ... ... ... [1884]
,, *Topographical and Sectional Anatomy of the Female Pelvis* ... ... ... ... ... ... ... ... ... ... 1885
**Hart and A. H. F. Barbour, D. B.** *Manual of Gynecology.* (5) 3rd Ed. 1886
**Harvey, W.** ... *Works.* (Tr. by R. Willis.) ... ... ... (5, 23) 1847

**Harvey, W.** ... *On Deafness* ... ... ... ... ... ... ... (25) 2nd Ed. 1856
,, *On Rheumatism, &c., in relation to Deafness* ... (25) [1857]
**Hassall, A. H.** *On Urine* ... ... ... ... ... ... ... ... ... (25) 1859
**Hasse, C. E.** ... *Pathological Anatomy.* (Tr. and Ed. by W. E. Swaine) ... ... ... ... ... ... ... ... ... (5) 1846
**Haultain and J. H. Ferguson, F. W. N.** *Handbook of Obstetric Nursing* ... ... ... ... ... ... ... ... ... (16) 1889
**Haüy, J. B.** ... *Anfangsgründe der Physik* ... ... ... ... ... ... (5) 1804
**Haüy, R. J.** ... *Handbuch der Physik* ... ... ... ... ... ... ... (5) 1805
**Heath, C.** ... *Minor Surgery and Bandaging* ... ... (25) 4th Ed. 1870
,, [Ed.] *Dictionary of Practical Surgery.* 2 vols. 3rd Ed. 1889
**Hebra, F.** ... *Diseases of the Skin.* 5 vols. (5) Vol. I. (Tr. and Ed. by C. H. Fagge) 1866; (5) Vol. II. (Tr. and Ed. by C. H. Fagge and P. H. Pye-Smith) 1868; (5) Vols. III.–V. (Tr. and Ed. by W. Tay), 1874, 1875, 1880
**Hecker, J. F. C.** *Epidemics of the Middle Ages.* (Tr. by B. G. Babington) ... ... ... ... ... ... ... ... (5) 1844
**Herschell, G.** ... *Health Troubles of City Life* ... ... ... (5) 2nd Ed. 1890
**Hewer, Mrs. L.** *Our Baby* ... ... ... ... ... ... ... ... ... ... ... 1891
**Hewson, W.** ... *Works.* (Ed. by G. Gulliver) ... ... ... ... (5) 1846
**Hill, B.** ... ... *The Essentials of Bandaging* ... ... (5) 6th Ed. 1887
**Hilles, M. W.** ... *The Anatomist* ... ... ... ... ... ... ... ... (23) 1860
**Hippocrates (Cos.)** *Genuine Works.* 2 vols. (Tr. and Ed. by F. Adams) ... ... ... ... ... ... ... ... ... (5) 1849
**Hirst, B. C.** [Ed.] *System of Gynecology and Obstetrics.* Obstetrics 4 vols. 1889
**Hoblyn, R. D.** ... *Dictionary of Medical Terms* ... ... ... ... 11th Ed. 1887
**Hoffmann, F.** ... *Opera Omnia.* 6 vols. ... ... ... ... ... ... ... 1754–61
**Holland, H.** ... *Chapters on Mental Physiology* ... ... ... ... (25) 1852
**Holmes and J. W. Hulke, T.** [Eds.] *A System of Surgery.* 3 vols. 3rd Ed. 1883
**Holt, B.** ... ... *Stricture* ... ... ... ... ... ... ... (25) 2nd Ed. 1863
* **Home, Sir E.** ... *Lectures on Comparative Anatomy.* (5) Vols. II., III., V., VI. ... ... ... ... ... ... ... ... 1814–28
,, *On Tumours* ... ... ... ... ... ... ... ... ... ... (19) 1830
**Hood, W. P.** ... *On Bone-setting* ... ... ... ... ... ... ... ... (25) 1871
**Hooker, W.** ... *Physician and Patient.* (Ed. by E. Bentley.) (25) 1850
**Hooper, R.** ... *Medical Dictionary* ... ... ... ... ... ... (5) 5th Ed. 1825
**Hulke, T. Holmes and J. W.** [Eds.] *A System of Surgery.* 3 vols. 3rd Ed. 1883
**Humphry, G. M.** *The Human Skeleton* ... ... ... ... ... ... ... (10) 1858
**Hunt, T.** ... ... *On the Skin* ... ... ... ... ... ... ... (25) 8th Ed. 1865
**Hunter, J.** ... *A Treatise on the Venereal Disease* ... (5) 2nd Ed. 1788
,, *Essays and Observations.* (Ed. by R. Owen.) 2 vols. 1861
**Hunter, W.** ... *Anatomia Uteri Humani Gravidi*—Latin and English. (7) 1774
**Hussey, E. L.** ... *Miscellanea Medico-Chirurgica.* (23) 1882; (5) Part II. 1887
,, *In Accidents—and at Other Times* ... (5) 3rd Ed. 1889
**Hutchinson, J.** *On the Spirometer* ... ... ... ... ... ... ... ... (25) 1852

**Hutchinson, J.** *The Pedigree of Disease* ... ... ... ... ... ... (15) 1884
,, *Syphilis* ... ... ... ... ... ... ... ... ... ... ... (5) 1887
**Hutchinson, P. S.** *Diseases of the Nose and Throat* ... ... ... ... ... 1891

*Influenza, Annals of* (Ed. by T. Thompson) ... ... ... ... (5) 1852

**Jacobi, A.** ... *Intestinal Diseases of Infancy and Childhood* ... (16) 1887
**Jaksch, R. v.**... *Clinical Diagnosis.* 2nd Ed. (Tr. by J. Cagney.) (16) 1890
**James, P.** ... *Guide to B.P.* ... ... ... ... ... ... (16) 3rd Ed. 1889
,, *The Therapeutics of Kronenquelle Water* ... ... (5) 1889
**Jamieson, W. A.** *Diseases of the Skin* ... ... ... ... ... ... (16) 1888
**Jenner, E.** ... *The Causes and Effects of the Variolæ Vaccinæ*—
(5) 3rd Ed. 1801
**Johnson, G.** ... *The Harveian Oration*, 1882 ... ... ... ... (3) 1882
,, *A Defence of Harvey* ... ... ... ... ... ... ... (3) 1884
,, *On Asphyxia* ... ... ... ... ... ... ... ... (5) 1889
**Johnson, J.** ... *The Influence of Tropical Climates on European Constitutions.* (3) 3rd Ed. 1821.
(30) 6th Ed. (conjointly with J. R. Martin) 1841
**Jones, H. B.** ... *The Chemistry of the Urine* ... ... ... ... ... (5) 1845
**Jones, H. M.** ... *Noises in the Head and Ears*... ... ... ... ... ... 1891
**Jones, T. W.** ... *On the Sense of Vision* ... ... ... ... ... ... (25) 1851

**Keating, J. M.** [Ed.] *Cyclopædia of the Diseases of Children.* 8 vols. 1889-91
**Keetley, C. B.**... *Index of Surgery* ... ... ... ... ... (16) 4th Ed. 1887
**Keith, S.**... ... *On Galvanism* ... ... ... ... ... ... ... ... [N.D.]
**Kennedy, E.** ... *On Pregnancy and Auscultation* ... ... .. ... (25) 1833
**Kerckringius, T.** *Spicilegium Anatomicum* ... ... ... ... ... (3) 1670
**Kirkes, W. S.**... *Handbook of Physiology* ... ... ... (23) 7th Ed. 1869
* **Klebs, E.** ... *Die Allgemeine Pathologie* ... (5) Zweiter Theil 1889
* **Klein, E.**... ... *Anatomy of the Lymphatic System* ... ... (5) I. 1873
,, *Elements of Histology* ... ... ... (5) [4th Ed.] 1889
**Kölliker, A.** ... *Manual of Human Histology.* (Tr. and Ed. by G. Busk and T. Huxley.) 2 vols. ... ... 1853-54
**Kramer, W.** ... *On Diseases of the Ear.* (Tr. by H. Power) (5) 1863
* **Küchenmeister, F.** *Manual of Parasites.* 2nd Ed.
(5) Vol. I. (Tr. by E. Lankester) 1857
**Kussmaul and A. Tenner, A.** *On Epileptiform Convulsions from Hæmorrhage.* (Tr. by E. Bronner) ... ... (5) 1859

**Laennec, R. T. H.** *Traité de l'Auscultation médiate.* 2 vols. (3) 2nd Ed. 1826
**Lancereaux, E.** *Treatise on Syphilis.* (Tr. by G. Whitley.) 2 vols. (5) 1868-69
**Landolt, E.** ... *The Refraction and Accommodation of the Eye.*
(Tr. by C. M. Culver) ... ... ... ... ... (21) 1886
**Lane, H.**... ... *Differentiation in Rheumatic Diseases* (*so called.*) (28) 1890
**Langerhans, P.** *Handbuch für Madeira* ... ... ... ... ... ... (5) 1885

**Latham, P. M.** *Clinical Medicine* ... ... ... ... ... ... ... (25) 1836
,, *Diseases of the Heart.* (23) Vol. I. 2nd Ed. 1846; (23) Vol. II. 1846
,, *Works.* (Ed. by R. Martin.) 2 vols. ... (5) 1876–78
**Latham, P. W.** *On the Formation of Uric Acid in Animals* ... (5) 1884
**Law, J.** ... ... *Captain Lobe* ... ... ... ... ... ... ... ... ... (5) 1889
**Lawrence, W.** .. *Lectures on Man* ... ... (25) 1822. (25) 6th Ed. 1834
,, *Lectures on Surgery*... ... ... ... ... ... ... (25) 1829-30
**Lee, E.** ... ... *Invalid's Companion to the Continent.* (5) 2nd Ed. 1861
**Lee, R.** ... ... *On the Diseases of Women* ... ... ... ... ... ... (19) 1833
,, *Hysteria* ... ... ... ... ... ... ... ... ... ... (30) 1871
**Lee, T. S.** ... *On Tumours of the Uterus* ... ... ... ... ... (25) 1847
**Lewers, A. H. N.** *Diseases of Women* ... ... (5) 1888. 3rd Ed. 1891
**Lewis, T. R.** ... *Physiological and Pathological Researches* ... ... (5) 1888
*Library of the New York Academy of Medicine, Periodicals, &c., in the*— Part I., United States and Great Britain ... (18) 1889
*Library of the Royal College of Surgeons of England, List of Transactions, &c., in the* ... ... ... ... ... ... ... 1890
*Life Assurance Companies* ... ... ... ... ... ... ... ... ... ... (3) 1872
**Lindley, J.** ... *Introduction to the Natural System of Botany* ... (5) 1830
**Liston, R.** ... *Elements of Surgery* ... ... ... ... ... ... (25) [N.D.]
,, *Operative Surgery.* (26) 2nd Ed. 1838. (5) 3rd Ed. 1840
*London, A Handbook to*... ... ... ... ... ... ... ... ... ... ... ... 1891
**Longney, E.** ... *Medical Lexicon* ... ... ... ... ... ... ... ... (16) 1888
**Louis, P. C. A.** *Recherches sur la Phthisie* ... ... ... ... ... ... (3) 1825
,, *Researches on Phthisis.* 2nd Ed. (Tr. by W. H. Walshe) (5) 1844
**Lower, R.** ... *Tractatus de Corde* ... ... ... ... ... ... ... ... (5) 1669
**Lumley, W. G.** *The New Lunacy Acts* ... ... ... ... ... ... ... (19) 1845

**M.D.** ... ... *Rhyming and Mnemonic Key to Materia Medica*... ... [N.D.]
**Macalister, A.** .. *A Text-Book of Human Anatomy* ... ... ... ... 1889
**Mackenzie, G. H.** *The Sputum* ... ... ... ... ... ... ... ... ... ... ... [1886]
**Mackenzie, M.**... *Hay Fever* ... ... ... ... ... ... ... (5) 3rd Ed. 1885
**Mackness, J.** ... *Dysphonia Clericorum* ... ... ... ... ... ... ... (25) 1848
**Maclagan, T. J.** *Fever* ... ... ... ... ... ... ... ... ... ... ... (5) 1888
**Mahony, O'B.**... *Cholera* ... ... ... ... ... ... ... ... ... ... (25) 1853
**Macnamara, C. M. Tidy and R.** *The Maybrick Trial* ... ... ... (7) 1890
**Malgaigne, J. F.** *Operative Surgery.* (Tr. by F. Brittan.) (10, 30) 1846
**Mann, M. D.** (Ed.) *System of Gynecology and Obstetrics.* Gynecology, 4 vols. ... ... ... ... ... ... ... ... ... ... 1889
*Manuel pratique de la Garde-Malade, &c.* (5) Tome I. 4th Ed. 1889; (5) Tome II. 1889; (5) Tome III. 4th Ed. 1889; (5) Tome IV. 4th Ed. 1889; (5) Tome V. 1888
**Marshall, A. M.** *The Frog*... ... ... ... ... ... ... (5) 3rd Ed. 1888
**Marston, J. A.** *Typhoid Fever and Tropical Life* ... ... ... ... (28) 1890

Martin, J. Johnson and J. R. *Tropical Climates* ... ... (30) 6th Ed. 1841
Martindale, W. *The Extra Pharmacopœia* ... ... ... (16) 6th Ed. 1890
Martinet, L. ... *Pathology.* (Tr. by J. Quain) ... ... (19) 2nd Ed. 1827
" *Manuel de Clinique.* 2 vols. ... ... ... ... (5) 1835
Massey, G. B.... *Electricity in Diseases of Women* ... ... ... 2nd Ed. 1890
Maxwell, T. ... *Terminologia Medica Polyglotta* ... ... ... ... ... 1890
Mayne, R. G.... *Medical Vocabulary.* 6th Ed. (Ed. by W. W. Wagstaffe.) 1889
M'Bride, P. ... *A Guide to the Study of Ear Disease* ... ... ... ... [1884]
McVail, J. C. ... *Vaccination Vindicated* ... ... ... ... ... ... (16) 1887
Meadows, B. ... *Eruptions* ... ... ... ... ... ... ... (16) 5th Ed. 1870
" *The Errors of Homœopathy* ... ... (23) 3rd Ed. 1876
Millar, J. ... *Hints on Insanity* ... ... ... ... ... ... ... (25) 1861
Millard, H. B. *Bright's Disease* ... ... ... ... ... ... ... (15) 1884
Milne, A. ... *Principles and Practice of Midwifery.* (30) 2nd Ed. 1879
Mitchell, Kate *The Drink Question* ... ... ... ... ... ... (5) [N.D.]
Mitchell, T. R. *On the Use of the Speculum* ... ... ... ... ... (25) 1849
Money, A. ... *Practice of Medicine* ... ... ... ... ... ... (16) 1889
" *Treatment of Disease in Children* ... ... (16) 2nd Ed. 1890
Monro, H. ... *On Insanity* ... ... ... ... ... ... ... ... (5) 1851
Moore, C. H. ... *On Going to Sleep* ... ... ... ... ... ... ... (25) 1868
Moore, N. ... *Visceral New Growths* ... ... ... ... ... ... (5) 1889
Morgagni, J. B. *De Sedibus et Causis Morborum* ... ... ... ... (8) 1761
Moullin, C. W. M. *Surgery* ... ... ... ... ... ... ... ... ... ... (27) 1891
Moure, E. J. ... *Amygdalotomie et Hémorragie* ... ... ... ... ... 1891
Müller, J. ... *Embryology with the Physiology of Generation.* (Tr. by W. Baly) ... ... ... ... ... ... (23) 1848
Munk, W. ... *Roll of the Royal College of Physicians.*— 3 vols. (20) 2nd Ed. 1878
Munnicks, J. ... *De Re Anatomica* ... ... ... ... ... ... ... ... 1697
Murphy, E. W. *Chloroform in Childbirth* ... ... ... ... ... (25) 1855
Murray, W. ... *Inductive Method in Medicine* ... ... ... ... ... 1891
Murrell, W. ... *Chronic Bronchitis* ... ... ... ... ... ... ... (16) 1889

Napier, A. D. L. *The Thermometer in Obstetrics and Gynecology* ... ... 1890
Neale, R. ... *The Medical Digest.* (5) 1877. (8, 28) 2nd Ed. 1882.
*The Medical Digest and Appendix.* (16) 1882–86.
*The Medical Digest.* 1840—90 ... ... 3rd Ed. 1891
Neligan, J. M. *Medicines: their Uses and Mode of Administration.* (23) 4th Ed. 1854
Neubauer and J. Vogel, C. *On the Urine.* 4th Ed. (Tr. by W. O. Markham) ... ... ... ... ... (5) 1863
Niemeyer, F. v. *Lectures on Phthisis.* 2nd Ed. (Tr. by C. Bæumler.) (5) 1870
Nittinger, C. G. G. *Die Impfregie mit Blut und Eisen, &c.* ... ... (3) 1868
Nixon, C. J. ... *Hospital Practice and Physical Diagnosis* ... ... ... 1889
*Nomenclature of Diseases, The* ... ... ... (30) 1869. (30) 2nd Ed. 1885
Nunneley, T. ... *Anatomical Tables* ... ... ... ... ... ... ... (23) 1838

**Oesterlen, F.** ... *Medical Logic.* (Tr. and Ed. by G. Whitley.) (5) 1855
**Oliver, G.** ... *The Harrogate Waters* ... ... ... ... ... ... ... (5) 1881
**Oliver, T.** ... *Lead Poisoning* ... ... ... ... ... ... ... ... 1891
**Orfila, P.**... ... *Leçons de Médecine légale.* 2 vols. ... ... (3) 1823–26
**Owen, E.** ... *A Manual of Anatomy* ... ... ... ... ... ... ... 1890
**Owen, R.** ... *On the Homologies of the Vertebrate Skeleton* ... (5) 1848
,, *On the Nature of Limbs* ... ... ... ... ... (5) 1849

**Paget, Sir J.** ... *Lectures on Surgical Pathology* ... ... (5) 4th Ed. 1876
**Parey, A.** ... *Works.* (Tr. by T. Johnson) ... ... ... ... (8) 1649
**Paris, J. A.** ... *Pharmacologia.* 2 vols. ... ... ... (19) 6th Ed. 1825
**Parker, L.** ... *On Syphilitic Diseases* ... ... ... ... (25) 4th Ed. 1860
**Parkes, E. A.**... *Cholera* ... ... ... ... ... ... ... ... ... ... (30) 1847
**Parkes, L. C.** ... *Hygiene and Public Health* ... ... ... (16) 2nd Ed. 1890
* **Parry, C. H.** ... *Elements of Pathology and Therapeutics.* (5) Vol. I. 2nd Ed. 1825
**Paulus** (Ægineta) *Seven Books.* (Tr. by F. Adams.) 3 vols. (5) 1844–47
**Pavy, F. W.** ... *The Harveian Oration,* 1886... ... ... ... ... (3) [N.D.]
* **Paxton, J.** ... *Introduction to the Study of Human Anatomy.* (5) Vol. I. 1834
**Payne, J. F.** ... *Manual of General Pathology* ... ... ... ... (5) 1888
**Peacock, T. B.**... *Malformations of the Heart* ... ... ... (5) 2nd Ed. 1866
**Pemberton, C. R.** *Diseases of the Abdominal Viscera* ... (19) 4th Ed. 1820
**Pepper, A. J.** ... *Elements of Surgical Pathology* ... ... (5) 2nd Ed. 1884
*Pharmacopœia, British* ... ... ... ... ... ... ... ... ... (30) 1867 1885
*Pharmacopœia, British.* Additions ... ... ... ... ... ... ... ... 1890
**Philip, R. W.**... *Pulmonary Tuberculosis* ... ... ... ... ... ... ... 1891
**Phillips, C. D. F.** *Materia Medica and Therapeutics: Vegetable Kingdom,* 1874; *Inorganic Substances* ... ... ... ... ... ... (15) 1882
**Piper, R. U.** ... *Operative Surgery Illustrated* ... ... ... ... (25) 1852
**Plinius, C.** (secundus) *Naturalis Historia* ... ... ... ... 3 vols. (8) 1669
**Pollock, J. E.**... *The Harveian Oration,* 1889 ... ... ... ... ... (3) 1889
**Pollock and J. Chisholm, J. E.** *Medical Handbook of Life Assurance.* 3rd Ed. 1889
**Porritt, N.** ... *Intra-thoracic Effusion* ... ... ... ... ... ... (5) 1883
**Pott, P.** ... ... *Works.* (Ed. by J. Earle.) 3 vols.... ... ... (5) 1790
**Potter, S. O. L.** *Compend of Anatomy* ... ... ... ... ... ... (5) 1887
**Power, V. D. Harris and D.** *Physiological Laboratory.* (28) 4th Ed. 1888
**Praagh, W. V.** *Oral Instruction of the Deaf and Dumb* ... ... (5) 1884
**P[richard], A.**... *Crosby Leonard* ... ... ... ... ... ... ... ... (23) [N.D.]
* **Prichard, J. C.** *Researches into the Physical History of Mankind.*— (5) Vol. I. 3rd Ed. 1836
**Pritchard, U.**... *Diseases of the Ear* ... ... ... ... ... ... 2nd Ed. 1891
**Prochaska, J. A. Unzer and G.** *On the Nervous System.* (Tr. and Ed. by T. Laycock) ... ... ... (5) 1851
**Prout, W.** ... *On Stomach and Renal Diseases* ... ... (5) 5th Ed. 1848
**Purdy, C. W.** ... *Diabetes* ... ... ... ... ... ... ... ... ... ... 1890

**Quain, J.** ... *Anatomy.* 2 vols. (5) 8th Ed. (Ed. by W. Sharpey, A. Thomson, and E. A. Schäfer.) 1876. 10th Ed. (Ed. by E. A. Schäfer and G. D. Thane.) Vol. I., Part I., II., 1890-91; Vol. II., Part I. ... ... ... ... ... ... ... 1890
**Quain, R.** ... *The Harveian Oration,* 1885... ... ... (5) 2nd Ed. 1886

**Radicke, —** .. *The Application of Statistics to Medical Enquiries.* (Tr. by F. T. Bond) ... ... ... ... (3, 5) 1861
**Ratier, F. S.** ... *Formulaire des hôpitaux de Paris* ... ... (3) 2nd Ed. 1825
**Rees, G. O.** ... *On Diseases of the Kidney* ... ... ... ... ... (25) 1850
**Reeves, H. A.**... *Human Morphology: The Limbs and the Perinæum.* (10) 1882
*Registration of the Causes of Death* ... ... ... ... ... ... ... (6) 1845
**Rendall, S. M.** *The Waters and Baths of Aix-les-Bains* ... ... (5) 1887
**Rentoul, R. R.** *The Causes and Treatment of Abortion* ... ... (5) 1889
**Rhazes, —.** ... *On the Small-Pox and Measles.* (Tr. by W. A. Greenhill) ... ... ... ... ... ... ... ... (5) 1848
**Richards, J. M.** *A Chronology of Medicine* ... ... ... ... ... (24) 1880
**Rigby, E.** ... *On Uterine Hæmorrhage* ... ... ... (25) 4th Ed. 1789
**Rigby, E.** ... *Midwifery* ... ... ... ... ... ... ... ... (23) 1844
,, *Dysmenorrhœa* ... ... ... ... ... ... (25, 30) 1844
**Rilliet, A. C. E. Barthez et F.** *Maladies des Enfants.* 3 vols. ... ... 1843
**Rindfleisch, E.**.. *Pathological Histology.* (Tr. by E. B. Baxter.) 2 vols. ... ... ... ... ... ... ... ... (5) 1872-73
**Ringer, S.** ... *Handbook of Therapeutics* .. ... (16) 12th Ed. 1888
**Roberton, J.** ... *Physiology and Diseases of Women, and on Midwifery.* (25) 1851
**Roberts, F. T.**... *The Officinal Materia Medica*... ... ... (5) 2nd Ed. 1887
,, *The Theory and Practice of Medicine*— (5) 6th Ed. 1885. (28) 7th Ed. 1888
,, *Notes on Additions to the B.P.* ... ... ... ... ... ... 1891
**Roberts, W.** ... *Urinary and Renal Diseases* ... ... ... ... ... (5) 1865
**Rodet, P.** ... *Vittel* ... ... ... ... ... ... ... ... ... ... (5) 1887
**Rohé, G. H.** ... *Text-Book of Hygiene* ... ... ... (11) 2nd Ed. 1890
**Rokitansky, C.** *Pathological Anatomy.* 4 vols. (5) Vol. I. (Tr. by W. E. Swaine), 1854; (5) Vol. II. (Tr. by E. Sieveking), 1849; (5) Vol. III. (Tr. by C. H. Moore), 1850; (5) Vol. IV. (Tr. by G. E. Day) ... ... ... ... ... ... ... ... 1852
**Romberg, M. H.** *Diseases of the Nervous System.* (Tr. and Ed. by E. H. Sieveking.) 2 vols. ... ... ... ... (5) 1853
**Roose, R.**... ... *Nerve Prostration* ... ... ... ... ... ... ... (28) 1888
,, *Gout*... ... ... ... ... ... ... ... ... (16) 6th Ed. 1889
**Ryan, M.** ... *Manual of Midwifery* ... ... ... ... (25) 3rd Ed. 1831

**Sansom, A. E.** ... *Lettsomian Lectures* ... ... ... ... ... ... (5) 1883
* **Santos, R. D.** ... *Clinique obstétricale* ... ... ... ... (5) Tome I. 1886
**Satterthwaite, T. E.** [Ed.] *A Manual of Histology* ... ... ... (28) 1881
**Saundby, R.** ... *Lectures on Bright's Disease* ... ... ... ... (5) 1889
,, *Lectures on Diabetes* ... ... ... ... ... ... ... ... 1891
**Sawyer, Sir J.** ... *Practical Medicine* ... ... ... ... ... ... ... 2nd Ed. 1891
**Sayre, L. A.** ... *Spinal Disease and Curvature* ... ... ... ... (23) 1877
**Schleiden, T. Schwann and M. J.** *Researches.* (Tr. by H. Smith).. (5) 1847
**Schneer, J.** ... *Alassio* ... .. ... ... ... ... ... ... ... ... (5) 1887
**Schroeder van der Kolk, J. L. C.** *On the Spinal Cord.* (Tr. by W. D. Moore) ... ... ... ... ... ... ... ... (5) 1859
,, *Atrophy of the Brain.* (Tr. by W. D. Moore.) (3, 5) 1861
**Schwann and M. J. Schleiden, T.** *Researches.* (Tr. by H. Smith.) (5) 1847
**Scudamore, C.** ... *On Gout, Gravel, and Rheumatism* ... (5) 3rd Ed. 1819
*Selected Monographs* ... ... ... ... ... ... ... ... ... ... .. (5) 1861
**Semple, R. H.** ... *Diseases of the Heart* ... ... ... ... ... ... (23) 1875
**Senn, N.** ... ... *Principles of Surgery* ... ... ... ... ... ... (27) 1890
**Sewill, H.** ... *Dental Surgery* ... ... ... ... ... ... (16) 3rd Ed. 1890
**Seymour, E. J.** *On Dropsy* ... ... ... ... ... ... ... ... ... (25) 1837
**Shaffer, N. M.** . *What is Orthopædic Surgery?* ... ... ... ... ... ... 1890
**Shaw, J.** ... ... *Anatomy* ... ... ... ... ... ... ... ... ... (10) 2nd Ed. 1822
**Sheen, A.** ... *Workhouse Medical Officer* ... ... ... (5) 2nd Ed. 1890
**Shoemaker, J. V.** *Diseases of the Skin* ... ... ... ... ... ... ... (5) 1888
,, *Heredity, Health and Personal Beauty* ... ... ... (27) 1890
* ,, *Materia Medica, Pharmacology and Therapeutics.* Vol. II. ... ... ... ... ... ... ... ... ... ... ... 1891
,, *Ointments and Oleates* ... ... ... ... ... ... 2nd Ed. 1890
**Silk, J. F. W.** ... *Nitrous Oxide Anæsthesia* ... ... ... ... ... ... (5) 1888
**Silvester, H. R.** *The True Physiological Method of Restoring Persons apparently Drowned or Dead* ... ... ... ... (25) 1858
**Simon, J. F.** ... *Chemistry.* (Tr. and Ed. by G. E. Day.) 2 vols. (5) 1845–46
**Simpson, Sir J. Y.** *Works.* 3 vols. Vol. I., Obstetrics. (Ed. by J. W. Black); Vol. II., Anæsthesia. (Ed. by Sir W. G. Simpson); Vol. III., Diseases of Women. (Ed. by A. R. Simpson) ... ... 1871
**Sims, J. M.** ... *Clinical Notes on Uterine Surgery* ... ... ... ... (8) 1866
**Skey, F. C.** ... *Ulcer* ... ... ... ... .. ... ... ... ... ... (10, 23) 1837
**Smedley, J.** ... *Practical Hydropathy* ... ... ... ... (5) 14th Ed. 1872
**Smellie, W.** ... *Obstetric Plates* ... ... ... ... (30) 2nd Ed. 1761
,, *Midwifery.* (Ed. by A. H. McClintock.) 3 vols. (5, 28) 1876–78
**Smith, F. W.** ... *The Saline Waters of Leamington* ... (5) 2nd Ed. 1885
**Smith, H.** ... *The Surgery of the Rectum* ... ... ... (25) 3rd Ed. 1871
**Smith, N.** ... *Curvatures of the Spine* ... ... ... ... (23) 2nd Ed. 1888
,, *Spasmodic Wry-Neck* ... ... ... ... ... ... ... ... 1891

**Smith, S.** ... *Principles and Practice of Operative Surgery.* 2nd Ed. 1887
**Smith, W. T.** ... *The Periodoscope* ... (25) 1848
**Snow, H.** ... *Proclivity of Women to Cancer* ... 1891
**South, J. F.** ... *The Craft of Surgery* ... (8) 1886
**Sozinskey, T. S.** *Medical Symbolism* ... 1891
**Spratt, G.** ... *Obstetric Tables.* 2 parts ... (9) 2nd Ed. 1835
**Squire, B.** ... *Diseases of the Skin* ... (25) 4th Ed. 1887
**Squire, P.** ... *Three Pharmacopœias* ... (25) 1851
,, *Companion to the British Pharmacopœia.* (5) 12th Ed. (conjointly with P. W. Squire and A. H. Squire) 1880
**Starr, L.** ... *Diseases of the Digestive Organs in Children.* 2nd Ed. 1891
**Steavenson, W. E.** *Spasmodic Asthma* ... (5) 1879
**Steggall, J.** ... *A Manual for Apothecaries' Hall* ... (19) 5th Ed. 1831
**Stone, W. H.** ... *The Harveian Oration,* 1887 ... (3) 1887
**Stricker, S.** [Ed.] *Human and Comparative Histology.* (Tr. by H. Power). 3 vols. ... (3, 5) 1870–73
**Styrap, J. de** ... *The Young Practitioner: his Code and Tariff* ... 1890
*Surgeon's Vade-Mecum, The* ... (25) 3rd Ed. 1824
*Surgery, Memoirs of the Academy of.* (Tr. and Ed. by D. Ottley.) (5) 1848
**Sutherland, H.** *Directory of Justices in Lunacy for* 1890-91 ... (5) 1890
**Swan, J.** ... *Demonstration of the Nerves* ... (23) 1834
**Swanzy, H. R.** *Handbook of Diseases of the Eye* ... (5) 1884
**Swedenborg, E.** *The Brain* ... (10) Vol. I. 1882
**Sydenham, T.** ... *Opera Omnia.* (Ed. by W. A. Greenhill) ... (5) 1844
,, *Works.* (Tr. by R. G. Latham.) 2 vols. (5) 1848–50
**Syme, J.** ... *On Stricture of the Urethra, &c.* ... (25) 1849
,, *Observations in Clinical Surgery* ... (30) 1861
,, *Excision of the Scapula* ... 1864

**Tamplin, R. W.** *On Curvature of the Spine* ... (25) 1852

**Tanner, J.** ... *Practical Midwifery, including Anæsthetics* ... (30) 1871

**Tanner, T. H.** ... *Clinical Medicine* ... (5) 1855

,, *Index of Diseases* ... (5) 1866

**Taylor, A. S.** ... *Medical Jurisprudence.*— (30) 2nd Ed. 1846. (5) 5th Ed. 1854. (23) 6th Ed. 1858

,, *Poisons* ... (23) 2nd. Ed. 1859

**Tenner and A. Kussmaul, A.** *On Epileptiform Convulsions from Hæmorrhage.* (Tr. by E. Bronner) 1859

*Therapeutics and Materia Medica at Ninth International Congress* ... (23) 1887

**Thompson, Sir H.** *Tumours of the Bladder* ... (5) 1884

,, *Diseases of the Urinary Organs* ... (5) 8th Ed. 1888

,, *Modern Cremation* ... (5) 1889

**Thomson, A. T.** *Conspectus of the British Pharmacopœias.* (Ed. by E. L. Birkett) ... (23) 17th Ed. 1852

**Thornton, B.** ... *Comparative Climatology* ... 1891

**Thorowgood, J. C.** *Bronchial Asthma* ... ... ... ... (5) 3rd Ed. 1887
**Tibbits, H.** ... *Medical Electricity* ... ... ... ... ... ... (19) 1873
**Tidy and R. Macnamara, C. M.** *The Maybrick Trial* ... ... ... (7) 1890
**Tidy, J. E. P. Clarke and C. M.** *Medical Law for Medical Men* ... ... 1890
**Tilt, E. J.** ... *On Uterine and Ovarian Inflammation.* (30) 3rd Ed. 1862
**Titiano [Vecelli.]** *Notomie* ... ... ... ... ... ... ... ... ... [N.D.]
**Todd, R. B.** ... *Clinical Lectures: Nervous System* ... (25) 2nd Ed. 1856
**Todd and W. Bowman, R. B.** *Physiological Anatomy.*— 2 vols. Vol. I. 2nd Ed. 1856; Vol. II. 1859
**Tomes, J.** ... *Dental Physiology and Surgery* ... ... . ... (8) 1848
**Treves, F.** ... *Surgical Applied Anatomy* ... ... ... (5) 2nd Ed. 1884
,, [Ed.] *Manual of Surgery.* 3 vols.... ... ... ... ... (16) 1886
**Troeltsch, A. V.** *On Diseases of the Ear.* (Tr. by J. Hinton.) (5) 1874
* **Trousseau, A.**... *Clinical Medicine.* (10) Vol. I. (Tr. and Ed. by P. V. Bazire), 1868; (5) Vols. II.—IV. (Tr. by J. R. Cormack) ... ... ... ... ... ... ... 1869-71
**Tuke, D. H.** ... *Prichard and Symonds* ... ... ... ... ... ... ... . 1891
**Tuke, J. B.** ... *A Plea for the Scientific Study of Insanity* ... ... ... 1891
**Turner, D.** ... *Dissertation sur les Maladies vénériennes.* 2 vols. ... 1767
**Turner, E.** ... *Elements of Chemistry* ... ... ... ... (19) 4th Ed. 1833
**Tyndall, J.** ... *Natural Philosophy* ... ... ... ... ... ... (5) [N.D.]

**Underwood, M.** *On the Diseases of Children.* (25) 9th Ed. (Ed. by M. Hall) ... ... ... ... ... ... ... ... 1835
**Unzer and G. Prochaska, J. A.** *On the Nervous System.* (Tr. and Ed. by T. Laycock)... ... ... ... ... ... ... (5) 1851

*Vaccination Commission.* (16) 1st *Report,* 1889; (16) 2nd *Report* 1890
**Velpeau, A.** ... *Diseases of the Breast.* (Tr. by M. Henry) ... (5) 1856
**Vintras, A.** ... *Mineral Waters of France* ... ... ... ... ... (5) 1883
*Vocabulary, Lewis's Medical* ... ... ... ... ... ... ... ... ... 2nd Ed. 1891
**Vogel, J.** ... *Icones Histologiae Pathologicae*—Latin and German 1843
,, *Pathological Anatomy of the Human Body* ... ... (30) 1847
**Vogel and C. Neubauer, J.** *On the Urine.* 4th Ed. (Tr. by W. O. Markham) ... ... ... ... ... ... ... ... (5) 1863

**Wagner, A.** ... *The Resection of Bones and Joints.* (Tr. by T. Holmes) ... ... ... ... ... ... ... ... ... (5) 1859
**Wainewright, J.** *A Mechanical Account of the Non-Naturals.* (5) 2nd Ed. 1708
**Waller, C.** ... *On the Function and Diseases of the Womb* ... ... (25) 1841
**Walshe, W. H.** *On Cancer* ... ... ... ... ... ... ... ... ... (3) 1846
,, *Diseases of the Lungs and Heart* ... ... (23) 2nd Ed. 1854
**Ward, S. H.** ... *Life Assurance* ... ... ... ... ... ... ... (25) 1857

| | | |
|---|---|---|
| **Wardrop, J.** ... | *Diseases of the Heart* ... ... ... ... ... .. ... (23) | 1851 |
| **Waring, E. J.** | *Bibliotheca Therapeutica.* 2 vols. . ... ... (3, 5) | 1878–79 |
| **Warlomont, E.** | *Manual of Animal Vaccination.* (Tr. and Ed. by A. J. Harries) ... ... ... ... ... ... ... (16) | 1885 |
| **Waters, A. T. H.** | *Emphysema of the Lungs* ... ... ... ... ... (25) | 1862 |
| **Watson, T.** ... | *The Principles and Practice of Physic.* 2 vols. (5) 3rd Ed. | 1848 |
| **Watson, W. S.** | *Diseases of the Nose* ... ... ... ... (2) 2nd Ed. | 1890 |
| **Wedl, C.**... ... | *Pathological Histology.* (Tr. and Ed. by G. Busk) (5) | 1855 |
| **Weightman, H.** | *The Medical Practitioner's Legal Guide* ... ... (5) | 1870 |
| **West, C.** ... ... | *Diseases of Infancy and Childhood.* (23) 3rd Ed. | 1854 |
| ,, | *The Harveian Oration for* 1874 ... ... ... ... (3) | 1874 |
| *What to Observe* | ... ... ... ... ... ... ... ... ... ... ... ... (3) | 1853 |
| **Williams, C. T.** | *Influence of Climate in Pulmonary Consumption.* (5) | 1877 |
| **Williams, J. C.** | *On Diseases of the Heart* ... ... ... (25) 2nd Ed. | 1852 |
| **Willmott, W. B.** | *Glycerin and Cod Liver Oil* ... ... ... ... ... (25) | 1860 |
| **Wilson, E.** ... | *Diseases of the Skin* ... ... ... ... (5) 2nd Ed. | 1847 |
| **Wilson, G.** ... | *Handbook of Hygiene* ... ... ... ... (5) 3rd Ed. | 1877 |
| **Winckel, F.** ... | *Diseases of Women.* (Tr. by J. H. Williamson.) (5) | 1887 |
| ,, | *Text-Book of Midwifery.* (Tr. by J. C. Edgar) ... | 1890 |
| **Windle, B. C. A.** | *Surface Anatomy* ... ... ... ... ... ... ... ... (5) | 1888 |
| **Winslow, F.** ... | *Obscure Diseases of the Brain and Mind.* (23) 3rd Ed. | 1863 |
| **Wise, A. T.** ... | *Alpine Winter in its Medical Aspects.* (5) 3rd Ed. | 1886 |
| **Wolfe, J. R.** ... | *Original Contributions to Ophthalmic Surgery* ... (5) | 1890 |
| *Women, Diseases* | *Peculiar to.* (Ed. by F. Churchill) ... ... ... (5) | 1849 |
| **Woodhead, G. S.** | *Bacteria and their Products* ... ... ... ... ... ... | 1891 |
| **Wunderlich, C. A.** | *Medical Thermometry.* 2nd Ed. (Tr. by W. B. Woodman) ... ... ... ... ... ... ... (5) | 1871 |
| | | |
| **Yearsley, J.** ... | *On the Throat* ... ... ... ... ... ... ... ... (25) | 1842 |
| **Yeo, J. B.** ... | *Food in Health and Disease* ... ... ... ... ... (16) | 1889 |
| ,, | *The Treatment of Typhoid Fever* ... ... ... ... ... | 1891 |
| **Young, S.** ... | *The Annals of the Barber-Surgeons of London* ... ... | 1890 |
| | | |
| **Zacchia, P.** ... | *Quæstiones Medico-legales* ... ... ... .. ... (5) | 1651 |
| **Ziegler, E.** ... | *General Pathological Anatomy* ... ... ... 2nd Ed. | 1885 |
| ,, | *Special Pathological Anatomy.* Sect. I.—VIII. 2nd Ed. 1887. Sect. IX.—XII. ... ... ... | 1886 |

### TRANSACTIONS, REPORTS, JOURNALS, &c.

*The List of current Periodicals to be seen in the Library is given in the* Journal *for March.*

*Abstract of the Medical Sciences, Half-yearly—
(30) Vols. I.—III.; (17, 30) Vols. IV.—XVIII.;
(30) Vols. XIX.—LVII. ... ... ... ... ... ... 1845-73
American Journal of Ophthalmology, The. (10) Vols. I.—VII. 1885-90
*American Practitioner and News, The—
(27) Vols. I.—VIII. in four volumes ... ... ... 1886-89
*American Surgical Association, Transactions of the—
Vol. I., 1883; Vol. II. 1885
Analectic, The ... ... ... ... ... ... ... ... ... Vol. I. 1884
Continued as *The Medical Analectic.*
Annual of the Universal Medical Sciences. 5 vols. (16) 1888;
5 vols. (16) 1889; 5 vols. (4, 30) 1890; 5 vols. 1891
*Archiv für pathologische Anatomie und Physiologie und für
klinische Medicin ... ... ... ... ... ... ... (5) 1861
Army—
* Statistical Reports on the Sickness, &c., among the
Troops in the United Kingdom, &c. ... ... ... (3) 1853
*Army Medical Departments—Statistical, Sanitary and Medical
Reports for 1860, 1861, 1862. 3 vols. ... ... ... (3) 1862-64
*Association of American Physicians, Transactions of the—
(5) Vols. II., III. 1887-88
Association Medical Journal. (Continuation of *Provincial
Medical and Surgcal Journal*) ... ... ... ... ... ... ... (25) 1853-57
Continued as *British Medical Journal.*

*Bibliotheca Medico-Chirurgica... ... (31) 1889; (31) 1890; (31) 1891
Biennial Retrospect of Medicine and Surgery, A. (Continuation
of *A Year Book of Medicine and Surgery*) 5 vols.
(5) 1867; (5) 1869; (5) 1871; (5) 1873; (5) 1875
*Bristol Health Report, 1890 ... ... ... ... ... ... ... ... (11) 1891
Bristol Medico-Chirurgical Journal ... ... Vols. I.—VIII. 1883-90
Bristol Medico-Chirurgical Society, Transactions of the—
(10, 28) Vol. I. 1878
*Brain ... ... ... ... ... ... ... ... ... ... ... ... Vol. XIII. 1890

British and Foreign Medico-Chirurgical Review, The. 60 vols. (5) 1848-77
*British Gynæcological Journal, The—
Vols. II., III. 1887-88; Vols. V., VI. 1889-90
British Journal of Dermatology, The ... ... ... Vols. I., II. 1889-90
*British Medical Journal, The. (Continuation of *Association Medical Journal.*) (25) 1858-81; (5, 25) 1882-83; (25) 1884-89; Vol. II. 1890 ... .. ... ... ... ... ... ... ...Vol. I. 1891
British Medical Journal, Supplement to the ... 1890; Vol. I. 1891
British Nurses' Association—First Annual Report ... ... ... 1889

*Calendar of the Royal College of Surgeons of England ... (23) 1876
*Clinical Society's Transactions ... ... ... ... (15) Vol. X. 1877
*Collective Investigation Record, The ... ... (23) Vols. I., II. 1883-84

*Dentists Register ... ... ... ... ... ... ... ... ... (23) 1885
Directory of Second-hand Booksellers, The ... ... (16) 1888 1891
*Dublin Journal of Medical Science, The ... (5) Vol. LXII. 1881

*Edinburgh Medical Journal. (Combination of *Monthly Journal of Medical Science* and of *Edinburgh Medical and Surgical Journal.*) (5) Vols. II.—VII., 12 vols., 1857-62; (5) Vol. IX., 2 vols., 1864; (5) Vols. XII., XIII., 4 vols., 1867-68; (5) Vols. XVII.—XXI., 10 vols. ... ... ... ... ... ... ... ... ... ... ... 1872-76
*Epidemiological Society, Transactions of the (23) 1875-80; (5) 1883

*Glasgow Medical Journal, The. (27) Vols. XXI.—XXXII. 1884-89;
Vols. XXXIV., XXXV. 1890-91

Hospital, The ... ... ... ... ... ... ... ...Vols. I.—IX. 1887-91
Hospitals:
Birmingham, The General Hospital, 1863 ... ... (3) 1864
* Guy's Hospital Reports. (17) Series III., Vols. I.—XXV., 1855-81; (17) Vols. XLI.—XLV., 1883-88; Vol. XLVII. 1890
* Middlesex Hospital. Registrars' Reports ... ... ... 1889
* St. Bartholomew's Hospital Reports. (5) Vols. I.—XXII. 1865-86
* St. Thomas's Hospital Reports ... ... ... Vol. XIX. 1891
Hygiene and Demography, "The Lancet" Reports of the International Congress of, 1876 to 1889 ... ... ... ... ... ... 1891
Hygiene and Demography, "Public Health" Special Report of the International Congress of, 1891 ... ... ... ... ... ... 1891

*Illustrated Medical News, The. (5) Vols. I.—V., 1888-89; Jan. 4, 11, 25 ... ... ... ... ... ... ... ... ... ... ... 1890
*Index Medicus ... ... ... ... ... ... ... ...(27) Vol. XI. 1890
International Clinics ... ... ... ... ... ... ... ... Vol. I. 1891
*International Medical Congress, Transactions of the. 5 vols. (30) 1881

*Journal of Anatomy and Physiology, The ... (28) Vol. XII. 1878
Journal of Cutaneous and Genito-Urinary Diseases. (Continuation of *Journal of Cutaneous and Venereal Diseases.*) Vols. V.—VIII. 1887-90
*Journal of Cutaneous and Venereal Diseases ... ... Vol. IV. 1886
Continued as *Journal of Cutaneous and Genito-Urinary Diseases.*
Journal of Physiology, The ... ... ... ... (28) Vols. I.—X. 1878-89

*Laboratory Reports. Royal College of Physicians, Edinburgh. Vol. III. 1891
*Lancet, The. (5) Vol. VIII., 1825; (5) 1833-44; (5) 1857-60; (5) 1865; (5) 1867-70; (5) 1876-78; (5) 1882-83; ... Vol. I. 1891
*Local Government Board, Annual Reports of the ... ... (5) 1871
*London Medical and Surgical Journal, The— (5) Vols. I.—III., V.—VII. 1832-35
*London Medical Record, The ... ... (27) Vols. XII.—XIV. 1884-86
Continued as *The London Medical Recorder.*
*London Medical Recorder, The. (Continuation of *The London Medical Record*)... ... ... ... ... ... ... ... (27) Vols. I., II. 1888-89
*Lunacy, Reports of the Commissioners in. (5) 1850; (5) 1851; (5) 1852

*Marine-Hospital Service of the United States, Annual Report of the Supervising Surgeon-General of the ... ... ... ... (5) 1889
Medical Analectic, The. (Continuation of *The Analectic.*) Vols. II.—VI. 1885-89
Continued as *The Medical Analectic and Epitome.*
Medical Analectic and Epitome, The. (Continuation of *The Medical Analectic*) ... ... ... ... ... ... ... Vol. VII. 1890
*Medical Annual, The. (16) 1887; (16) 1888; (16) 1889; (16) 1890 1891
*Medical Chronicle, The ... ... ... (8, 27) Vol. I., 1884-85 (27) Vols. II.—X.; 1885-89; Vol. XIII. 1890-91
*Medical Council, Minutes of the General ... (5) Vol. XXIV. 1887
*Medical Directory, The. (16) 1870; (16) 1874; (6) 1875; (23) 1884; (6) 1885; (6) 1887; 1890 1891
*Medical Officer of Privy Council, Reports of the— (3) 6th Report, 1864; (3) 9th Report 1867
*Medical Record, The— (27) Vols. XXIV.—XXXVI., 1883-89. Vol. XXXVIII. 1890
*Medical Register, The ... ... ... ... ... ... (6) 1871; (23) 1885
*Medical Times, The— (5) Vols. XII.—XV., 1845-47; (5) Vols. XVII.—XXIV. 1848-51
Continued as *The Medical Times and Gazette.*
*Medical Times and Gazette, The. (Continuation of *The Medical Times.*) (5) Vols. XXV.—XL., 1852-59; (5) 1860-64; (5, 14) 1865-66; (5) 1867-69; (5, 14) 1870-73; (5) 1874-75; (5) Vol. I., 1876; (5) 1878 ... ... ... ... ... ... ... ... (14) 1881-85

*Medico-Chirurgical Transactions—
(5) Vols. XXXIII.—LXVI., 1850-83; (5) LXVIII.—LXXII., 1885-89; Index, Vols. I.—XXXIII., 1851; Index, Vols. I.—LIII 1871
*Monthly Journal of Medical Science, The. (5) Vols. IX.—XI., 1849-50.
(5, 17) Vols. XII.—XVII., 1851-53; (5) Vols. XVIII.—XX. 1854-55
Merged in *Edinburgh Medical Journal.*

Navy:
* Statistical Reports on the Health of the Navy, 1837-43. (3) Part III. 1854
* Statistical Report on the Health of the Navy, 1888. (5) 1889
* Statistical Abstract on the Health of the Navy, 1867-68. (5) 1868
*New York Academy of Medicine, Transactions of the. Series II.
(18) Vol. I., 1874; (18) Vol. II., 1876; (16) Vol. III., 1883; (18) Vol. IV., 1886; (18) Vol. V., 1886; (18) Vol. VI. 1890
*New York Medical Journal, The—
(27) Vols. XXXVIII.—L., 1883-89; Vols. LII., LIII. 1890-91

*Obstetrical Transactions. (8) Vols. I. — XII. 1860-71; (7) Vols. XIV.—XXVI. 1873-85

*Prisons, Report of the Directors of Convict, 1863 ... ... (3) 1864
*Progrès médical, Le ... ... ... ... ... ... ... Tome XIII. 1891
Provincial Medical and Surgical Association, Transactions of the.
(17, 25) Vols. I.—V., 1833-37; (25) Part I., Vol. VI., 1837; (17, 25) Part II., Vol. VI., 1838; (5, 17, 25) Vol. VII., 1839; (5, 25) Vols. VIII.—XIV., 1840-46; (5) Vol. XV., 1847; (25) Parts I., II., Vol. XVI.—XIX. ... ... ... ... ... ... ... 1848-53
*Provincial Medical and Surgical Journal. (25) Vol. II. 1841; (25) 1842-52
Continued as *Association Medical Journal.*
From Vol. II., 1842, to Vol. VII., 1844, it was called *Provincial Medical Journal.*

*Registrar-General of Births, Deaths, and Marriages in England, Reports of ... ... ... ... ... ... ... (3) 1846; 1856-62 1865
*Répertoire général d'Anatomie et de Physiologie pathologiques, et de Clinique chirurgicale ... ... ... ... Tomes I.—VI. 1826-28
*Ditto. Atlas.
*Retrospect of Medicine, Braithwaite's. (25) Vol. I., 2nd Ed., 1841; (5) Vols. IV.—XLVIII., 1841-64; (25) Vols. LV.—LVII., 1867-68; (5, 25) Vol. LVIII., 1869; (25) Vols. LIX., LX., 1869-70; (5, 25) Vols. LXI.—LXIII., 1870-71; (5) Vols. LXIV. — LXX., 1872-74; (5) Vol. LXXIII., 1876; (5) Vol. LXXXV. 1882
*Revue médicale, La ... ... ... ... ... ... ... ... (5) Tome II. 1860
*Royal Academy of Medicine in Ireland, Transactions of the. Vol. VIII. 1890

*St. Andrew's Medical Graduates' Association, Transactions of the ... ... ... ... ... ... ... ... (6) Vols. V., VI. 1872-74

*Therapeutic Gazette, The ... ... ... ... Vols. VIII.—XIV. 1884-90

*Union médicale, L' ... ... ... ... 3rd Series. Tome LI. 1891

*West London Medico-Chirurgical Society, Transactions of the— (5) Vol. II. 1887

*Würzburger medicinische Zeitschrift. ... ... ... (5) 3 vols. 1861-2-3

*Year-Book of Medicine and Surgery, A. (5) 1861; (5) 1862; (5) 1863; (5) 1864; (5) 1865

Continued as *A Biennial Retrospect of Medicine and Surgery.*

*Year-Book of Scientific and Learned Societies ... ... 1890 1891

*Year-Book of Treatment, The ... ... ... ... ... ... (23) 1889 1891

*Zeitschrift der k.k. Gesellschaft der Arzte in Wien ... ... (5) 1864

*The Honorary Librarian,* Mr. L. M. Griffiths, *will be glad to receive, at* 28 *Berkeley Square, Bristol, any donations for the Library.*

*Contributions towards completing sets of those marked * will be gratefully accepted, and later Editions of many books are much desired.*

---

### SCRAPS

### PUBLICATIONS RECEIVED.

From Baillière, Tindall & Cox, London:
*Noises in the Head and Ears.* By H. Macnaughton Jones, M.D. 1891.
*Stertor and Apoplexy.* By Robert L. Bowles, M.D. 1891.

From C. Birchall, Liverpool:
*Discovery.* No. 6. Vol. I. June 1st, 1891.

From Geo. du Boistel & Co., Bristol:
*Bristol Health Report,* 1890. 1891.

From Breitkopf und Härtel, Leipzig:
*Über neue medicinische Seifen.* Von P. J. Eichhoff, M.D. 1890. (Reprint in English.)

From Ev. E. Carreras, St. Louis:
*The Alienist and Neurologist.* April, July, 1891.

From Cassell & Company Limited, London:
*The Treatment of Typhoid Fever.* By J. Burney Yeo, M.D. 1891.

From *El Censor,* Buenos Aires:
*Boletin de Sanidad Militar.* No. 5. 1891.
*Anales de la Asistencia Publica.* May—July, 1891.

From J. & A. Churchill, London:
*Prichard and Symonds.* By D. Hack Tuke, M.D. 1891.

From T. & A. Constable, Edinburgh:
*Atlas of Clinical Medicine.* By Byrom Bramwell, M.D. Vol. I. Parts I., II. 1891.

From Cornish Brothers, Birmingham:
*Pra.tical Medicine.* By Sir James Sawyer, Kt., M.D. Second Edition. 1891.

From Down Brothers, London:
*Ophthalmic Charts.* By Mr. Frank Haydon.

From Feret et Fils, Bordeaux:
*Annales de la Policlinique.* No. 5. 1891.

From Harper & Brothers, New York:
*Harper's Weekly.* May 16th, 1891.

From *The Journal of Mental Science,* London:
*Some Unusual Cases of General Paralysis.* By Bonville Bradley Fox, M.D. Reprint.

From J. N. Klingelfuss y Ca. Buenos Aires:
*Anales del Departmento Nacional de Higiene.* Nos. 3—5. 1891.

From H. K. Lewis, London:
*Diseases of Women.* By Arthur H. N. Lewers, M.D. Third Edition. 1891.
*Inductive Method in Medicine.* By William Murray, M.D. 1891.
*Painful Menstruation.* By Francis Henry Champneys, M.D. 1891.
*The British Journal of Dermatology.* July—September, 1891.
*Diseases of the Ear.* By Urban Pritchard, M.D. Second Edition. 1891.
*Differences in the Nervous Organisation of Man and Woman.* By Harry Campbell, M.D. 1891.

From Young J. Pentland, Edinburgh:
*A Plea for the Scientific Study of Insanity.* By J. Batty Tuke, M.D. 1891.
*International Clinics.* Edited by John M. Keating, M.D.; J. P. Crozer Griffith, M.D.; J. Mitchell Bruce, M.D.; David W. Finlay, M.D. Volume First. 1891.
*Lead Poisoning.* By Thomas Oliver, M.D. 1891.

From G. P. Putnam's Sons, New York:
*A Case of Intracranial Neoplasm.* By C. A. Oliver, M.D. 1891. Reprint.

From John M. Richards, London:
*Medical Reprints.* June—August, 1891.

From Smith, Elder & Co., London:
*Spasmodic Wry-Neck.* By Noble Smith. 1891.

